# Critical Study on the Tube-to-Chip Luer Slip Connectors

**DOI:** 10.3389/fmedt.2022.881930

**Published:** 2022-05-31

**Authors:** Leire Etxeberria, Unai Aguilera, Pablo Garcia de Madinabeitia, Alberto Saez, Ane M. Zaldua, José L. Vilas-Vilela, Luis Fernández, Andreu Llobera

**Affiliations:** ^1^microLIQUID S.L, Arrasate-Mondragón, Spain; ^2^Leartiker S. Coop., Markina-Xemein, Spain; ^3^Macromolecular Chemistry Research Group (LabQuiMac), Department of Physical Chemistry, Faculty of Science and Technology, University of the Basque Country (UPV/EHU), Leioa, Spain; ^4^BC Materials, Basque Center for Materials, Applications and Nanostructures, UPV/EHU Science Park, Leioa, Spain

**Keywords:** LoC devices, microfluidic connectors, tube-to-chip connection, Luer slip connectors, useful life

## Abstract

Luer slip is one of the gold standards for chip-to-world interface in microfluidics. They have outstanding mechanical and operational robustness in a broad range of applications using water and solvent-based liquids. Still, their main drawbacks are related to their size: they have relatively large dead volumes and require a significant footprint to assure a leak-free performance. Such aspects make their integration in systems with high microchannel density challenging. To date, there has been no geometrical optimization of the Luer slips to provide a solution to the mentioned drawbacks. This work aims to provide the rules toward downscaling the Luer slips. To this effect, seven variations of the Luer slip male connectors and five variations of Luer slip female connectors have been designed and manufactured focusing on the reduction of the size of connectors and minimization of the dead volumes. In all cases, female connectors have been developed to pair with the corresponding male connector. Characterization has been performed with a tailor-made test bench in which the closure force between male and female connectors has been varied between 7.9 and 55 N. For each applied closure force, the test bench allows liquid pressures to be tested between 0.5 and 2.0 bar. Finally, the analysis of a useful life determines the number of cycles that the connectors can withstand before leakage.

## Introduction

Microfluidics and, more generally, Lab-on-a-chip (LoC) systems have broadly been used to develop analytical tools for pharmaceutical, life science, and diagnostics (1). To date, LoC has shown outstanding performance and integration level to develop complex laboratory procedures at the microscale while requiring minute amounts of sample varying in the μL-pL range (2–6). Despite such extremely promising features, currently, microfluidics has only very seldomly reached commercial exploitation besides niche applications. The reason behind this weak economic impact can arguably be attributed to the chip-to-world interconnection (CWI) between the LoC and the peripherals (tubes, pumps, valves, etc.) (7–10). Even though this need was already identified in 2003 by Lie et al. (11–13), to date it is still an unsolved engineering need. Conceptually speaking, a robust CWI should (1) be biocompatible and chemically inert, (2) have zero or minimal dead volume, (3) be easy to plug, (4) be removable and reusable, (5) be reliable at operational pressures, (6) be made using simple and low-cost techniques, and (7) be fully compatible with commercial tubing and fittings (2).

Currently, one of the gold standards for CWI is the Luer slip connector due to easy handling and compatibility with medical and laboratory instruments, such as needles, syringes, and cannulas (3, 14, 15). The reason being is the ability to withstand fluidic pressures >2 bar without leakages, but equally important is to be automatable with a simple actuation movement and relative low force and being of multiple uses (2, 16). Despite such outstanding properties, Luer connectors have also severe drawbacks, mainly related to their size, since they have relatively large dead volumes, and require a significant footprint to assure leak-free. This approach goes against dense packaging or multiplexing (17, 18). Furthermore, as most prototyping techniques are not compatible for Luer manufacturing, researchers have sought for alternatives to Luer, such as a barb or direct connection without interfaces (19). The common industrial approach is then to develop highly specific connectors compatible only with their respective peripherals. Thus, a universal, reliable CWI is yet to be presented, hampering the overall applicability of LoC for large-scale applications.

Steps toward monolithic integration of Luer connectors with LoC have been done by taking advantage of the huge development of 3D printing. This technology offers flexibility, design freedom, and rapid prototype manufacturing providing good solutions for small fabrication volumes (20–23). The introduction of this highly versatile technology has allowed the prototyping of arbitrary complex LoC with a broad range of materials. Specifically related to 3D printing of polymers, their outstanding properties in terms of lightweight, low cost, optical transparency, and in some cases chemical resistances are unmatchable by other materials (24–26). Still, 3D printing is a serial process that lacks the required throughput to be considered as a mass-production technology. Even though there has been some progress related to multi-nozzle/parallel printing, it still can be considered as a technology only suitable for small batches, and other technologies, such as injection molding, are preferred since it is reported to be the cheapest and fastest approach for massive fabrication of LoC (16, 27, 28).

In this work, we aim to visualize this need from a different perspective. While keeping the full compatibility with injection molding, seven different Luer slip male connectors and five different Luer slip female connectors have been designed aiming toward the reduction of the aforementioned drawbacks while assuring their compatibility (whenever possible). To complete the study of the different approaches for tube-to-chip connection, the analysis of the useful life of the connector has been performed to define the number of cycles between 0 and 2 bar that the connectors can withstand before leakage.

## Design and Working Principle

**Figure 1:**
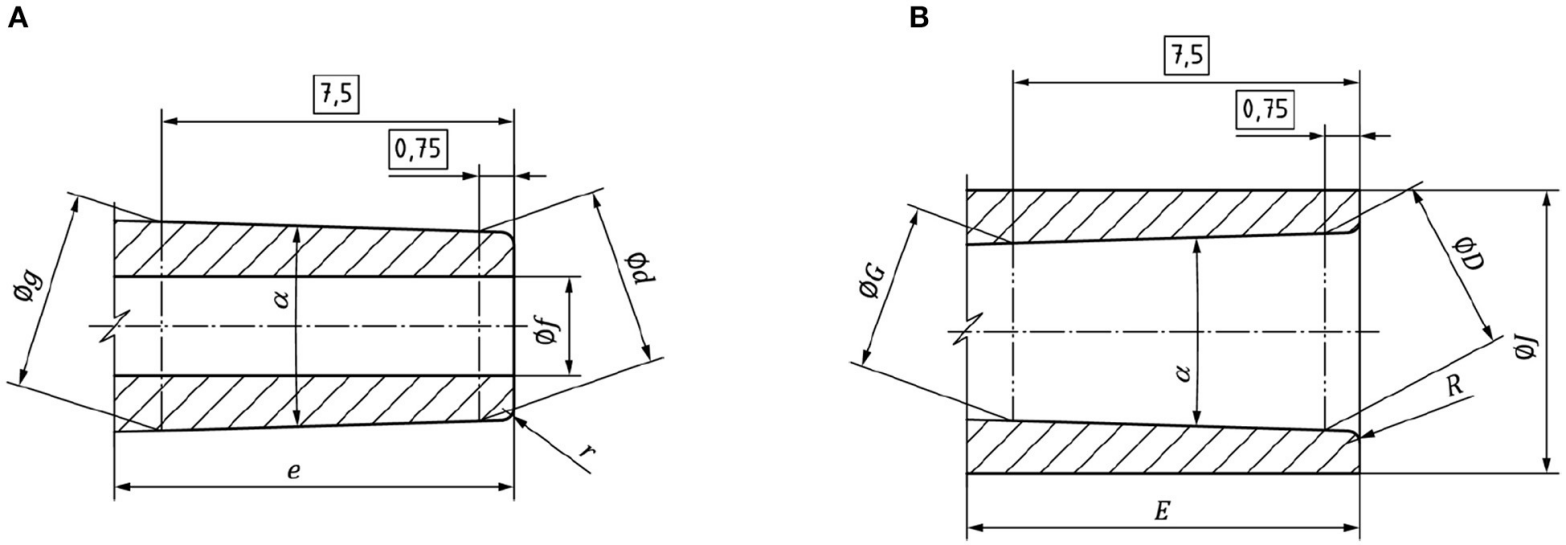
**(A)** Male and **(B)** female Luer connector drawings.

The proposed designs are based on the ISO 80369-7 (29) norm which defines the main parameters of the standard Luer connectors as shown in Figure 1. The male connector is described in Figure 1A, where α is the cone angle, Ød is the external diameter in the extreme of the male cone at 0.750 mm, *e* is the length of the cone, Øf is the internal diameter, Øg is the external diameter in the extreme of the male cone at 7.500 mm, and *r* is the extreme exterior cone radius. The female connector is described in Figure 1B, where Ø_D_ is the external diameter in the extreme of the female cone at 0.750 mm, *E* is the depth of the cone, Ø_G_ is the external diameter in the extreme of the female cone at 7.500 mm, Ø_J_ is the external diameter of the female connector, and *R* is the radius on the entrance of the female cone.

The working principle in Luer Slip connectors is based on the fitting between the external face of the male connector and the internal face of the female connector. The holding is performed by friction on both surfaces. Therefore, the height and inner diameters that affect the friction area as well as the dead volume will be the most critical parameters for the correct functioning of the connectors.

Considering those parameters as critical, Table 1 shows the new parameters proposed for both male and female designed connectors. Male 1 and Female 1 are the standard Luer connectors, which are considered the gold standards in this work. All dimensions have been downscaled to the half in Male 2 and three-fourths in Male 3. Minimization of the friction area between male and female connectors has been done by independently studying the different key regions of the Luer. Specifically, the connector height has been reduced to half and three-fourth in Male 4 and 5, while keeping the rest of the parameters unaltered. In Male 6 and Male 7, the inner diameter has been decreased to half and three-fourths, respectively, focusing on the reduction of dead volumes. In all cases, female connectors have been designed to pair with the corresponding male connectors. The compatibility between connectors is also summarized in Table 1.

**Table 1:** Description of the designs for male and female connectors in mm.

	**α**	**ød**	**e**	**øf**	**øg**	**r**
Male 1	3.440	4.021	8.400	2.100	4.426	0.250
Male 2		2.011	4.200	1.050	2.213	0.125
Male 3		3.016	6.300	1.575	3.320	0.188
Male 4		4.021	4.200	2.100	4.426	0.250
Male 5		4.021	6.300	2.100	4.426	0.250
Male 6		4.021	8.400	1.050	4.426	0.250
Male 7		4.021	8.400	1.575	4.426	0.250
	**α**	**øD**	**E**	**øG**	**øJ**	**r**
Female 1	3.440	4.248	8.400	3.843	6.356	0.250
Female 2		2.124	4.200	1.922	3.178	0.125
Female 3		3.186	6.300	2.882	4.767	0.188
Female 4		4.248	4.200	3.843	6.356	0.250
Female 5		4.248	6.300	3.483	6.356	0.250
**Compatibility**					
		**Pair (Perfectly match)**		**Compatible**	
Female 1		Male 1, 6 and 7		Male 4 and 5	
Female 2		Male 2		-	
Female 3		Male 3		-	
Female 4		Male 4		Male 1, 5, 6 and 7	
Female 5		Male 5		Male 1, 4, 6 and 7	

**Figure 2:**
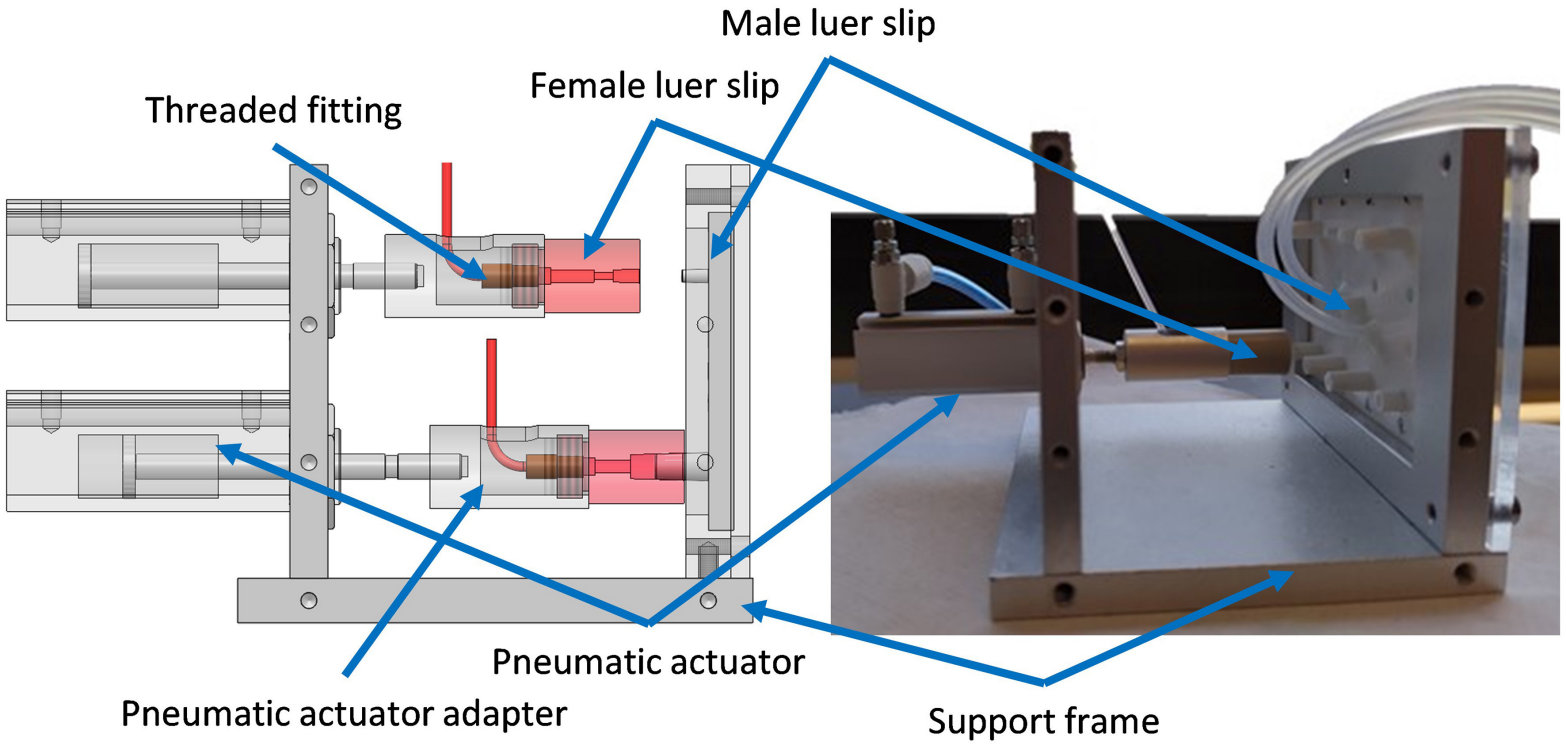
Images of the test bench including the chip with male connectors and female connectors in red.

Focusing toward standardization, a dedicated test bench has been built and is presented in Figure 2 and described in more detail in the Supplementary Material. The platform is composed of an aluminum base that holds for placing all the components in the correct position with fine alignment and distances. Thereby, the chip containing different male Luer slip connectors is placed on one side of the test bench. On the opposite side, a pneumatic actuator (CJP2B10-20D, SMC) can provide actuation forces larger than 55 N with response time (ON/OFF) in the millisecond range. In the resting position of the actuator, the connector is at a distance smaller than the maximum path of the actuator, so that when extended, both male and female connectors under study are interlocked. Switching off the pneumatic actuator assures returning to the initial position, allowing unloading of the measured pair.

**Figure 3:**
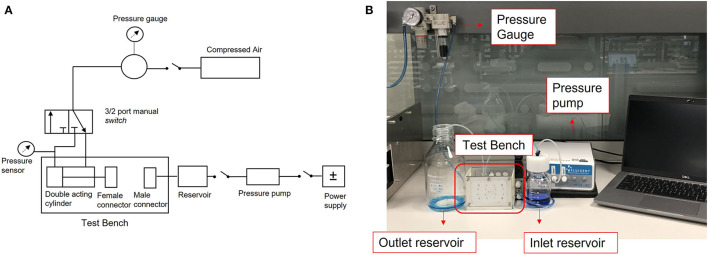
Experimental setup. **(A)** Schematic drawing of the test bench, **(B)** Image of the setup.

The actuation force is dependent on the input pressure; therefore, it can be modulated using a pressure regulator as observed in the schematic setup of the test bench, in Figure 3A. The obtained forces from applied pressure values provided by the datasheet are summarized in Table 2.

**Table 2:** Actuation force values.

**Pressure [bar]**	**Force [*N*]**
1	7.9 ± 0.5
2	15.8 ± 0.4
3	23.6 ± 0.4
4	31.5 ± 0.4
5	39.3 ± 0.4
6	47.2 ± 0.4
7	55.0 ± 0.4

When the female connector reaches the male connector, the perfusion of the liquid starts. The fluid is driven with a required fluidic pressure from the reservoir using the pressure pump Fluigent MFCS™-EZ. The tubing led the fluid to the female connector and once it matches the male connector, the fluid comes out through the outlet and is stored in a reservoir. The circuit has been connected to the connector using a commercial PEEK Threaded fitting for tube connections. Figure 3B shows the image of the complete experimental setup (see Supplementary Information for details).

## Materials and Methods

### Materials

**Figure 4:**
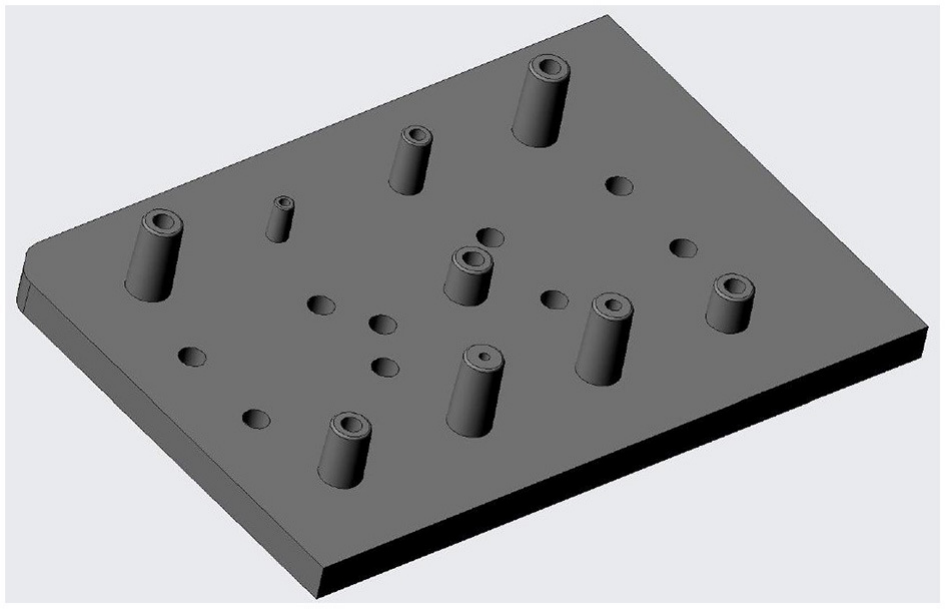
CAD image of the chip containing male connectors.

Male and Female connectors have been machined in different materials as the required properties vary. Male connectors are envisaged to be part of the LoC, and thus being single-use in most cases. Then, they have been machined in Ertalyte PET due to the dimensional stability, excellent wear resistance, and low coefficient of friction that offers. These properties make this material extremely suitable for the machining of high-precision parts (30). In addition, the designs can easily be transferred to injection molding technology. Figure 4 shows the CAD image of the chip containing male connectors.

On the other hand, female connectors have been machined in Ketron PEEK material. The resistance and biocompatibility of PEEK allow longer use of female connectors including the use of solvents and even biological samples (31).

### Methods

#### Machining of Connectors

The equipment used for the machining of the connectors is KONDIA B-1000 Machining center. The requirements for machining are summarized in Table 3 for male connectors and in Table 4 for female connectors. The defined tolerances were 0.05 mm in XY and 0.1 mm in Z. The machining of male connectors was performed in three steps. In the first step, the rotation speed of the spindle was 5,000 rpm with a feed rate of 200 mm/rev, the plunge depth (Ap) of 2 mm, and pass width (Ae) of 0.375 mm. In the second step, the rotation speed of 6,500 rpm and a feed rate of 950 mm/rev have been used for bulk machining. Here, the conditions of Ap and Ae were 8 and 0.15 mm, respectively. In the last step, a finishing machining was executed with a rotation speed of 7,000 rpm, feed rate of 1,000 mm/rev, and with Ap and Ae of 2 and 0.12 mm, respectively. For female connectors, the rotation speed of the spindle for the external machining process was 1,200 rpm with a feed rate of 0.15 mm/rev. In the case of the internal machining process, the rotation speed was 900 m/min with a feed rate of 0.25 mm/rev. In both cases, Ap and Ae were 500 μm and 1.5 mm, respectively.

**Table 3:** Machining requirements for Male connectors in Ertalyte.

**Male connectors**	**Height [mm]**	**External diameter (up) [mm]**	**External diameter (bottom) [mm]**	**Internal diameter [mm]**
Male 1	8.4 ± 0.1	4.12 ± 0.05	4.48 ± 0.05	2.10 ± 0.05
Male 2	4.2 ± 0.1	2.03 ± 0.05	2.22 ± 0.05	1.05 ± 0.05
Male 3	6.3 ± 0.1	3.08 ± 0.05	3.35 ± 0.05	1.57 ± 0.05
Male 4	4.2 ± 0.1	4.12 ± 0.05	4.23 ± 0.05	2.10 ± 0.05
Male 5	6.3 ± 0.1	4.12 ± 0.05	4.35 ± 0.05	2.10 ± 0.05
Male 6	8.4 ± 0.1	4.12 ± 0.05	4.48 ± 0.05	1.05 ± 0.05
Male 7	8.4 ± 0.1	4.12 ± 0.05	4.48 ± 0.05	1.58 ± 0.05

**Table 4:** Machining requirements for Female connectors in Ketron PEEK.

**Female connectors**	**Height in the connection area [mm]**	**Internal diameter connection area (Up) [mm]**	**Internal diameter connection area (Bottom) [mm]**	**External diameter [mm]**
Female 1	8.4 ± 0.1	3.79 ± 0.05	4.28 ± 0.05	11.50 ± 0.05
Female 2	4.2 ± 0.1	1.92 ± 0.05	2.17 ± 0.05	11.50 ± 0.05
Female 3	6.3 ± 0.1	2.40 ± 0.05	3.59 ± 0.05	11.50 ± 0.05
Female 4	4.2 ± 0.1	4.04 ± 0.05	4.28 ± 0.05	11.50 ± 0.05
Female 5	6.3 ± 0.1	3.91 ± 0.05	4.28 ± 0.05	11.50 ± 0.05

#### Characterization of Machined Parts

The dimensional control of the connectors was performed using a digital caliper for external diameter and height measurements and Nikon Eclipse 80i optical microscopy for internal diameter measurements. Moreover, 3D images of the connectors have been obtained using an optical profilometer ZYGO ZeGage Pro (Obj: 10X).

The fluidic tests have been performed using the setup described in Figure 3. Male-female closure has been checked between 7.9 and 55 N. For each applied closure force, the test bench allows liquid pressures to be tested between 0.5 and 2.0 bar. The upper-pressure limit is due to the pumping system (Fluigent MFCS™-EZ) and not on the selected pneumatic actuator.

Arguably, a fluidic pressure of 2.0 bar covers most of the microfluidic applications. Therefore, this fluidic pressure value has been defined as a final target in this work. The minimum required closing force at which the connector can withstand 2.0 bar of fluidic pressure without leakage has been analyzed. For each defined closing force, the number of cycles that the connectors last before leakage has been studied performing a useful life analysis. Using the test bench, the connectors have been actuated up to 100 times applying 2.0 bar fluidic pressure at defined closure force.

## Results and Discussion

### Dimensional Control of Machined Parts

**Figure 5:**
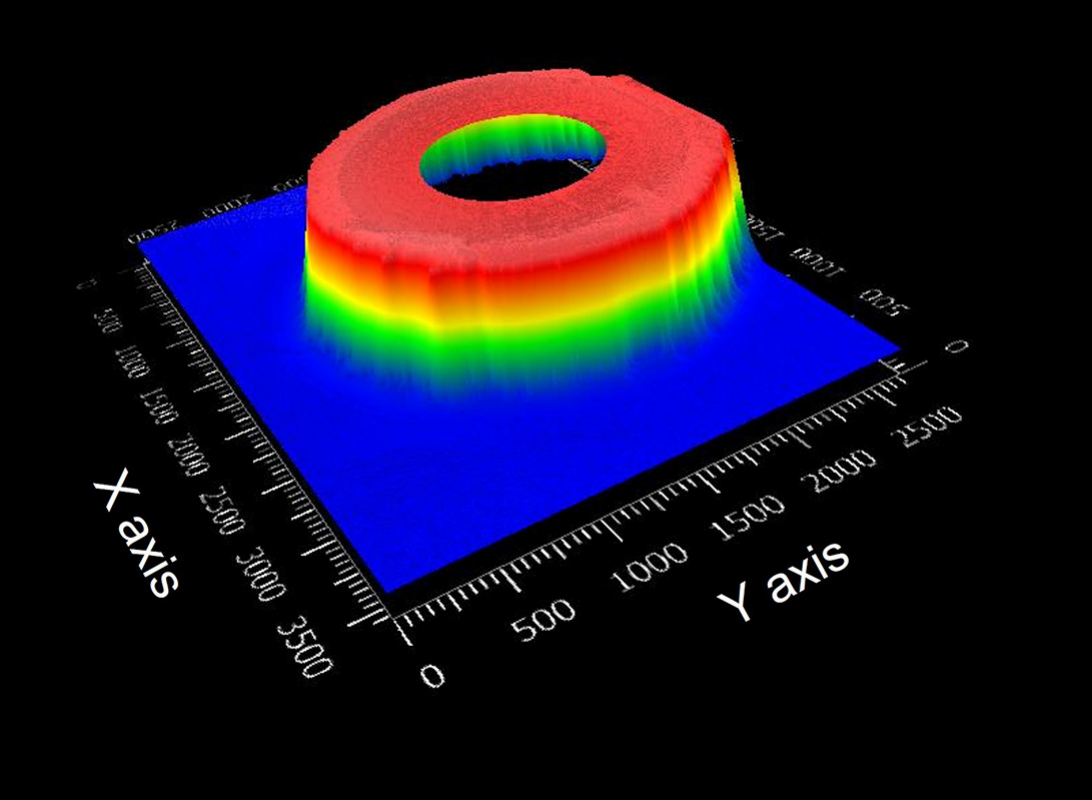
Optical profilometer image of a male connector.

The 3D image of the upper part of the male connector obtained using the optical profilometer is shown in Figure 5. Despite the visible machining marks on the outer wall of the connector, the picture shows a reliable image of the connector.

**Figure 6:**
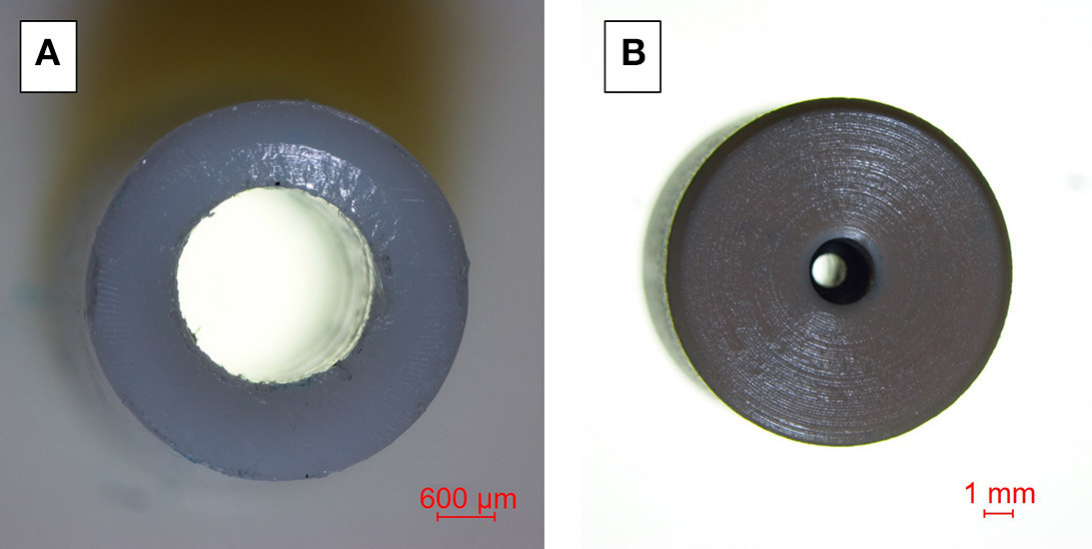
Images obtained with Nikon Eclipse 80i optical microscopy. **(A)** Male connector, **(B)** Female connector.

Figure 6 shows images obtained with the Nikon Eclipse 80i optical microscopy for dimensional control of machined parts. In this case, although the machining marks are more visible on the surface of the female connector, the male connector shows machining traces and thus, rougher finishing.

Tables 5, 6 present the measured dimensions of male and female connectors, respectively. In most of the cases, machined parts are according to the design specifications. The highest deviation can be observed in the internal diameter in female connectors. These deviations may be due to the measurement method and do not affect the overall characterization of the connectors.

**Table 5:** Measured dimensions of Male connectors.

**Male connectors**	**Height [mm]**		**External diameter (up) [mm]**		**External diameter (bottom) [mm]**		**Internal diameter [mm]**	
	**Meas**.	**Dev**.	**Meas**.	**Dev**.	**Meas**.	**Dev**.	**Meas**.	**Dev**.
Male 1	8.50	+0.1	4.10	−0.02	4.48	+0.01	2.10	+0.01
Male 2	4.17	−0.03	2.10	+0.07	2.24	+0.02	1.1	+0.05
Male 3	6.24	−0.06	3.12	+0.04	3.38	+0.03	1.73	+0.16
Male 4	4.19	−0.01	4.12	+0.01	4.27	+0.05	2.15	+0.05
Male 5	6.26	−0.04	4.12	+0.01	4.38	+0.03	2.08	−0.02
Male 6	8.47	+0.07	4.10	−0.02	4.51	+0.03	1.01	−0.04
Male 7	8.42	+0.02	4.09	−0.03	4.51	+0.03	1.63	+0.05

**Table 6:** Measured dimensions of Female connectors.

**Female connectors**	**Height in the connection area [mm]**		**Internal diameter connection area (Up) [mm]**		**Internal diameter connection area (Bottom) [mm]**		**External diameter [mm]**	
	**Meas**.	**Dev**.	**Meas**.	**Dev**.	**Meas**.	**Dev**.	**Meas**.	**Dev**.
Female 1	8.39	−0.01	3.76	−0.03	4.19	−0.09	11.54	+0.04
Female 2	–	–	–	–	2.17	+0.01	11.46	−0.04
Female 3	6.31	+0.01	–	–	3.53	−0.06	11.5	+0.01
Female 4	4.23	+0.03	4.07	+0.03	4.24	−0.04	11.49	−0.01
Female 5	6.31	+0.01	–	–	4.22	−0.06	11.47	−0.03

In conclusion, although machined parts show traces and marks typical of the process, the connectors are machined as specified and thus, have been considered as valid for this work.

### Fluidic Tests

Different combinations of male-female Luers have been tested—if compatible—and the results have been summarized in Table 7. Thereby, the minimum required closure force to achieve the goal is presented.

**Table 7:** Results for minimum closure force required to hold on 2.0 bar fluidic pressure for each possible connector combination.

**Connector type**	**Female 1**	**Female 2**	**Female 3**	**Female 4**	**Female 5**
Male 1	47.2 ± 0.4N	Not compatible	Not compatible	39.3 ± 0.4N	39.3 ± 0.4 N
Male 2	Not compatible	31.5 ± 0.4 N	Not compatible	Not compatible	Not compatible
Male 3	Not compatible	Not compatible	15.8 ± 0.4N	Not compatible	Not compatible
Male 4	23.6 ± 0.4 N	Not compatible	Not compatible	23.6 ± 0.4 N	47.2 ± 0.4N
Male 5	47.2 ± 0.4 N	Not compatible	Not compatible	2.0 bar fluidic pressure not reached	55.0 ± 0.4 N
Male 6	55.0 ± 0.4N	Not compatible	Not compatible	55.0 ± 0.4 N	55.0 ± 0.4N
Male 7	39.3 ± 0.4N	Not compatible	Not compatible	2.0 bar fluidic pressure not reached	47.2 ± 0.4 N

The results show that a reduction of height in the connectors (M4) is more suited at low connection force (above 25 N in its pair connector) due to the friction reduction between male and female parts. Contrarywise, reduction of internal diameter (M6, M7) causes an increase in the liquid velocity, making this more prone to leakage. From this data, it can be concluded that the best compromise is with M2 and M3, where an overall downscaling of all parameters has been done as compared to the gold standard and still retains its performance. This downscaling allows us to address the aforementioned discussion regarding the footprint and dead volumes, since it will allow a denser interconnection in advanced LoC.

In the theoretical case, leakage will only depend on the contact pressure between the connectors. Nevertheless, the length of the contact area and the surface finishing also affect the connector quality increasing the required closure force. The applied forces and the deformations in the material can also affect the quality of the connection. In this case, values of deformation and tensile stress at yield of both materials taken from the datasheets are much higher than expected experimental values, suggesting that the material will always work in the elastic region.

**Figure 7:**
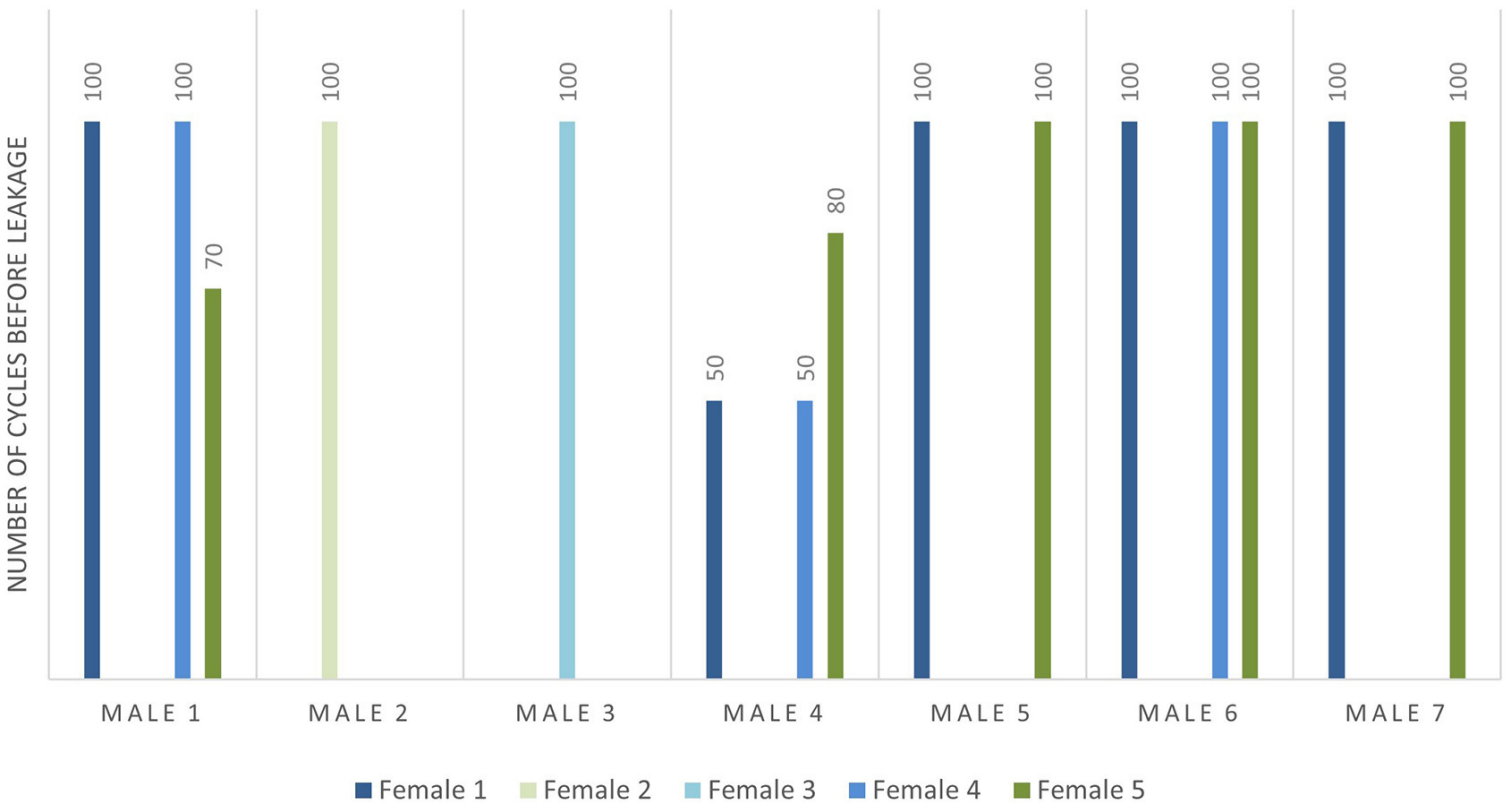
Number of cycles before leakage at 2 bar fluidic pressure for each possible connector combination.

Regarding the useful life of the connector, the analysis of the number of cycles before leakage with 2.0 bar of fluidic pressure plotted in Figure 7 shows that although the friction reduction between male and female (M4) needs a lower connection force, the number of cycles before leakage reduces considerably. The reduction of the internal diameter (M6, M7) and the downscaling (M2, M3) of the connectors do not affect the useful life of the connector.

It is therefore concluded that downscaled connectors (M2, M3) are the most reliable ones showing the best compromise between the dead volumes, due to their size and performance, requiring low closing forces, and lasting at least 100 cycles. Fluidic tests have been performed with 2 bar of fluidic pressure limited by the working range of the pressure pump. Although the defined maximum fluidic pressure is in the range of interest for microfluidic applications, the good behavior of the connectors in the tests suggests possible satisfactory performance also at higher fluidic pressure values.

## Conclusions

In this work, seven variations of the Luer slip male connectors and five variations of Luer slip female connectors have been designed, manufactured, and characterized to elucidate the strategies toward decreasing the footprint while retaining its performance. The main goal is the path to upscaling a reliable CWI in the LoC application.

Using a specifically designed and developed test bench, a minimum closure force required to hold on 2.0 bar fluidic pressure has been analyzed in which the downscaled connectors show the best compromise between the dead volume and closing force demanding 31.5 ± 0.4 N (M2) and 15.8 ± 0.4 N (M3). For the required closing force, a useful life analysis of the connectors has been performed by applying 100 closing cycles for each possible connector combination. Most of the compatible connectors withstand 100 cycles before leakage, except for the cases in which the friction area has been reduced (M4).

The study shows that the proportional downscaling of all dimensions in the standard Luer Slip connector allows the reduction of dead volumes without compromising the functionality of the connector. Furthermore, size reduction permits dense packaging or multiplexing in LoC devices and massive manufacturing of them using injection molding.

## Data Availability Statement

The original contributions presented in the study are included in the article/Supplementary Material, further inquiries can be directed to the corresponding authors.

## Author Contributions

LE, UA, PG, and AS contributed to the design and manufacturing of the connectors. UA prepared the CAD designs and the organization of the setup. LE performed the experiments and wrote the first draft of the manuscript. JV-V contributed to assessment during revisions. AZ, LF, and JV-V contributed with the founding and resources. AL contributed with the supervision and project administration. All authors contributed to manuscript revision, read, and approved the submitted version.

## Funding

The authors are grateful for the financial support from the Basque Country Government within the frame of the project BIKAINTEK 2018 (48-AF-W2-2018-00006).

## Conflict of Interest

LE, UA, PG, AS, LF, and AL were employed by microLIQUID S.L. LE and AZ were employed by Leartiker S. Coop. JV-V were employed by UPV/EHU and BC Materials. LE is a student of UPV/EHU.

## Publisher's Note

All claims expressed in this article are solely those of the authors and do not necessarily represent those of their affiliated organizations, or those of the publisher, the editors and the reviewers. Any product that may be evaluated in this article, or claim that may be made by its manufacturer, is not guaranteed or endorsed by the publisher.
