# Supplementary material for: Critical Study on the Tube-to-Chip Luer Slip Connectors
Source: Front Med Technol. 2022 May 31;4:881930. doi: 10.3389/fmedt.2022.881930 (PMC9194524; doi:10.3389/fmedt.2022.881930)
Supplement: Supplementary file 1 [file Data_Sheet_1.docx]

Supplementary Material

**Critical study on the tube-to-chip Luer slip connectors**

**L.Etxeberria^1,2,3*^, U. Aguilera^1^, P. Garcia de Madinabeitia^1^, A. Saez^1^, A.M. Zaldua^2^, José L. Vilas-Vilela^3,4^, L.Fernández^1^, A. Llobera^1^**

^1^ microLIQUID S.L, Goiru 9, 20500 Arrasate-Mondragon, Spain

^2^ Leartiker S. Coop., Xemein etorbidea 12, 48270 Markina-Xemein, Spain

^3^ Macromolecular Chemistry Research Group (labquimac), Department of Physical Chemistry, Faculty of Science and Technology, University of the Basque Country (UPV/EHU), Leioa, Spain

^4^ BC Materials, Basque Center for Materials, Applications and Nanostructures, UPV/EHU Science Park, Leioa, Spain

* Correspondence:
Leire Etxeberria
letxeberria@leartiker.com

# Test Bench design and images

Supplementary Figure 1. CAD image of Pneumatic actuation test bench


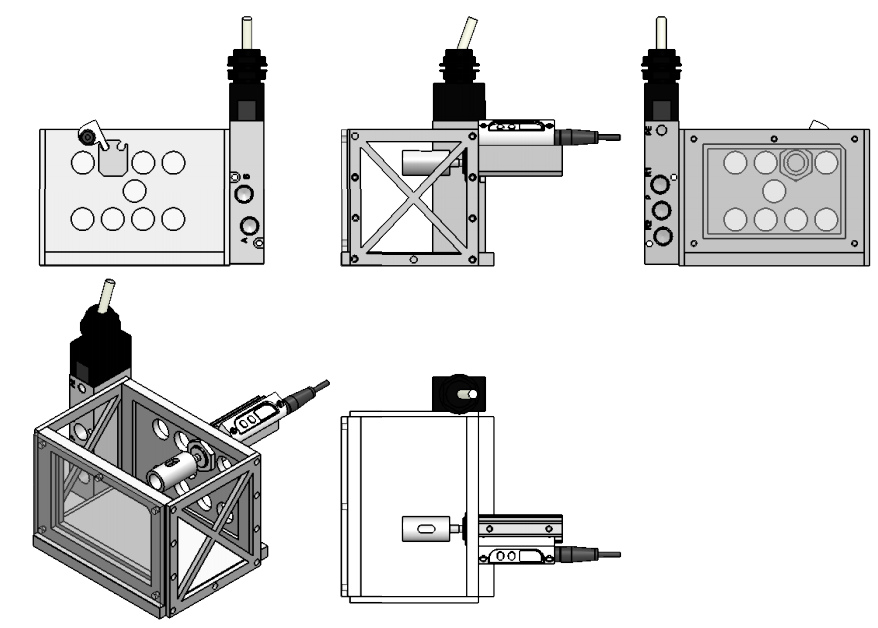


Supplementary Figure 2. Views of the test bench


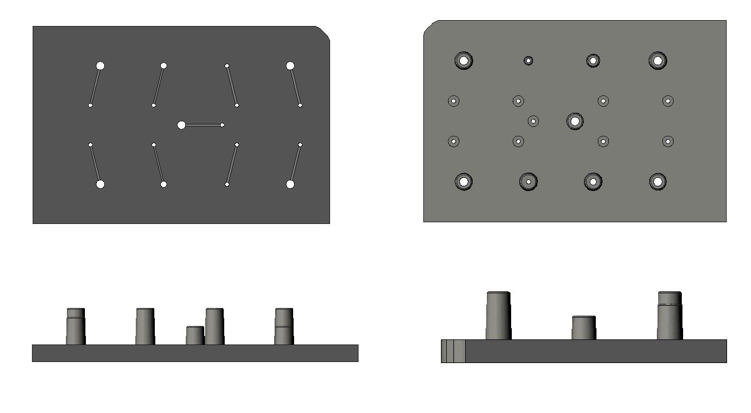


Supplementary Figure 3. Views of the chip containing male connectors


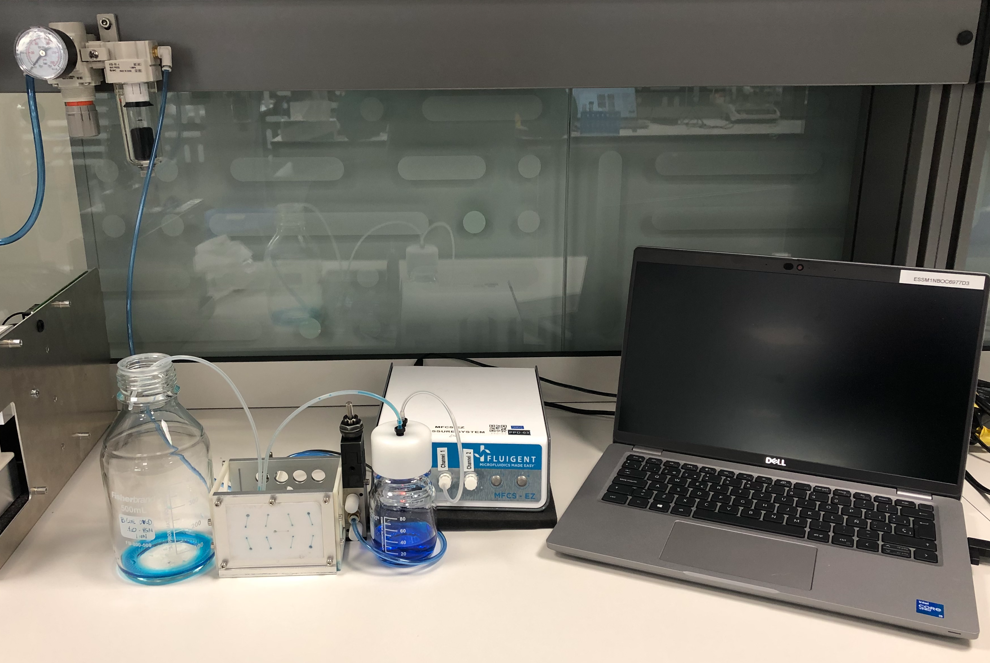


Supplementary Figure 4. Image of the complete setup


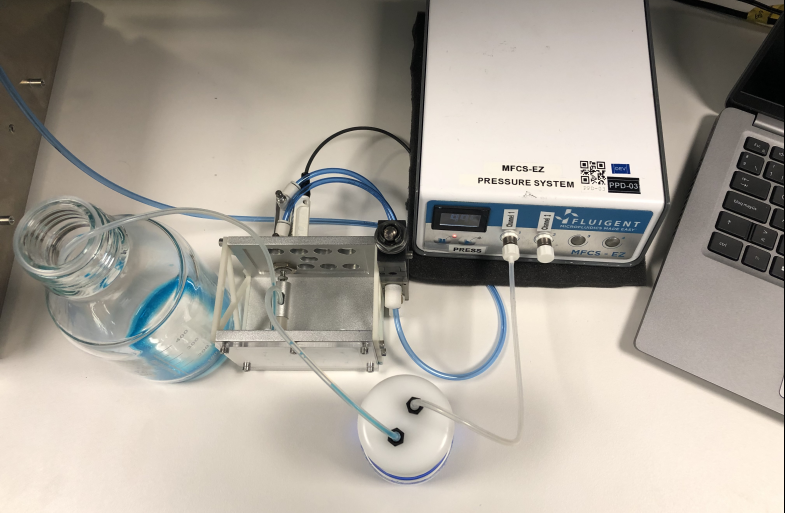


Supplementary Figure 5. Image of the setup (Pressure pump and test bench)


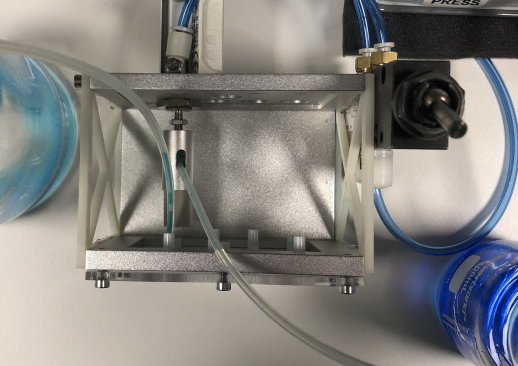


Supplementary Figure 6. Image of the setup (test bench)

# Test Bench components

Supplementary Table 1. List of components of the test bench

|  | Part Id | Description | Material | Quantity |
| --- | --- | --- | --- | --- |
| Mechanical parts | | | |  |
|  | 31011A | Base | Aluminium | 1 |
|  | 31021A | Actuator support | Aluminium | 1 |
|  | 31031A | Chip support | Aluminium | 1 |
|  | 31041A | Chip support cover | PMMA | 1 |
|  | 31051A | Support frame | Rigid resin | 1 |
|  | 31061A | Adapter | Aluminium | 1 |
| Commercial elements | | | |  |
|  | M3x5 DIN912 | Screw | INOX | 19 |
|  | CDJP2B10 | Pneumatic actuator | - | 1 |
|  | EVZM550-F01-08 | Mechanical switch | - | 1 |
|  | AN10-01 | Silencer | - | 2 |
|  | AS1201F-M3-04 | Flow regulator | - | 1 |
|  |  |  |  |  |
